# Values and trade-offs in responsible antibiotic behaviour and sustainable access: protocol for a comparative mixed-methods empirical ethics study in Sweden, Italy, and Bulgaria

**DOI:** 10.1186/s12910-026-01651-9

**Published:** 2026-11-03

**Authors:** Josepine Fernow, Niklas Juth, Antonia Diukendjieva, Gergana Kyosovska, Deborah Mascalzoni, Roberta Biasiotto, Jorien Veldwijk, Mirko Ancillotti

**Affiliations:** 1 Centre for Research Ethics & Bioethics (CRB), Department of Public Health and Caring Sciences, Uppsala University, Box 564, Uppsala, Sweden; 2 Bulgarian Association for Personalized Medicine (BAPEMED), Sofia, Bulgaria; 3 Institute for Biomedicine, Eurac Research, Bolzano, Italy; 4 Erasmus School of Health Policy & Management, Erasmus University Rotterdam, Rotterdam, The Netherlands

**Keywords:** Antibiotic resistance, Antibiotic stewardship, Sustainable access, One Health, Empirical ethics, Discrete choice experiment, Mixed methods, Social norms

## Abstract

**Background:**

Antibiotic resistance arises through biological evolution but is also shaped by behavioural, sociocultural, and institutional factors. Promoting responsible antibiotic behaviour requires understanding how members of the public and relevant stakeholders perceive antibiotic use, stewardship, access, and responsibility, and the values and trade-offs that shape their decisions. This study aims to investigate these factors across three national contexts and to develop context-sensitive recommendations and a methodological framework for future research.

**Methods:**

This protocol describes an ongoing comparative mixed-methods empirical ethics study conducted in Sweden, Italy, and Bulgaria from May 2025 to April 2028. The study comprises three connected components: semi-structured online interviews with 16–20 participants from four stakeholder groups in each country; a general-population survey incorporating a discrete choice experiment, with a minimum of 350 respondents per country; and iterative normative analysis using reflective equilibrium. The 3D Value Framework informs the interviews, which examine stakeholders’ reasoning about sustainable antibiotic access, stewardship, responsibility, and preparedness. Interview findings, relevant literature, and previous research will inform the survey attributes and scenarios. The survey will examine public preferences and trade-offs concerning antibiotic treatment, responsible behaviour, and sustainable access, alongside antibiotic knowledge, trust, prosociality, biospherism, gender-related perspectives, social norms, and values. The empirical findings will be interpreted in relation to ethical theories of justice and personal and collective responsibility. A One Health perspective informs the overall study.

**Discussion:**

By bringing stakeholder reasoning and public preferences into dialogue with normative analysis, the study will examine how responsibility for preserving antibiotic effectiveness and sustainable access should be understood and distributed between individuals and institutional actors. It will also address the ethical implications of trade-offs between present and future access and the distribution of associated benefits and burdens. This empirical ethics approach is expected to support a context-sensitive account of justice and responsibility and to inform ethically justified antibiotic stewardship, policy, and communication.

**Clinical trial number:**

Not applicable.

## Background

Antibiotic resistance (AR) arises through evolutionary processes in microorganisms, but its emergence and spread are not merely biological phenomena. They occur in a societal context, influenced by norms, values, behaviour and sociocultural factors that differ between countries [1]. The inappropriate use of antibiotics contributes to a major global public health challenge associated with substantial and increasing mortality. In 2019, an estimated 1.27 million deaths worldwide were attributable to bacterial antimicrobial resistance (AMR) [2]. Preventing unnecessary antibiotic use requires a good understanding of the determinants of socially responsible antibiotic behaviour, including how individuals understand the interdependence between individual and community health and the effects their behaviour can have on public health, especially in the case of infectious diseases. Effective antibiotic stewardship must respond to the social, institutional, and practical conditions in which antibiotic-related decisions are made. Communication is one component and is more likely to be effective when it engages with recipients’ norms, values, and circumstances. This, in turn, requires that both the public and global health dimensions of AR are studied and communicated clearly [3].

Understanding how behaviour and sociocultural factors influence antibiotic-related practices is crucial for short-term infection control and for ensuring sustainable and equitable access to effective antibiotic treatment. Moreover, this knowledge is crucial for designing long-lasting preventive measures and for informing policy and communication on responsible antibiotic use. Although knowledge is consistently regarded as a prerequisite for appropriate antibiotic use and for other behaviours affecting antibiotic resistance, such as willingness to vaccinate, travel choices, hygiene practices, and conscientious food consumption, we know that informing people is insufficient to modify their behaviour [4, 5]. Researchers have called for international cooperation and approaches that account for the social, cultural, and institutional contexts in which antibiotic-related behaviour occurs [6]. The design and viability of good AR stewardship programs depend on the contexts in which they are implemented. Moreover, the WHO has emphasised the importance of conducting context-sensitive research, including research on behavioural and sociocultural aspects [7].

At the same time, responsible antibiotic behaviour involves not only avoiding unnecessary use, but also preserving antibiotic effectiveness and ensuring access when antibiotics are needed, for both current and future patients. Antibiotic use has consequences beyond the individual patient because it can affect resistance patterns and the future effectiveness of treatment, while access to effective antibiotics is necessary for many other forms of healthcare [8]. Sustainable access concerns both present and future access and how benefits and burdens are distributed. Arrangements that secure access for present patients may weaken future access by reducing the conditions needed to maintain supply or by accelerating the loss of antibiotic effectiveness [9]. These challenges raise ethical questions about justice and about how responsibility for preserving antibiotic effectiveness and access should be understood and distributed at individual and collective levels [10].

Through a call focused specifically on Antimicrobial Resistance, the Swedish Research Council (Vetenskapsrådet) provided funding for an international collaborative project. The project, entitled ‘Humanities and social sciences in One Health: Promoting just and responsible antibiotic behaviour in society’, uses a mixed-methods approach to investigate how values, norms, and behavioural and sociocultural determinants influence antibiotic-related behaviour, stewardship, and sustainable access to effective antibiotics, across three national contexts. The project applies a One Health perspective and aims to explore the values, norms, and sociocultural determinants that shape stakeholders’ and the public’s antibiotic-related reasoning and decision-making; investigate the role of gender perspectives, trust, prosociality, biospherism, and other psychological constructs, social norms, and values to explain antibiotic behaviour; examine public preferences and trade-offs concerning antibiotic treatment, stewardship, and sustainable access to effective antibiotics; and develop a normatively robust conceptualisation of social justice and responsibility in the antibiotic resistance discourse through an iterative reflective-equilibrium process. The end result will be recommendations on how to use this knowledge to improve antibiotic stewardship programmes and, methodologically, a multi-layered blueprint protocol for investigating values, norms, and behavioural and sociocultural determinants of antibiotic resistance that can inform context-sensitive transnational research, policy strategies, and communication.

The project combines comparative qualitative interviews with relevant stakeholders, a survey of the general public incorporating a Discrete Choice Experiment (DCE), and an iterative normative analysis intended to support context-sensitive recommendations for research, policy, prevention, and communication. Sweden, Italy and Bulgaria were selected to enable comparison across different social and institutional contexts in countries with average or above-average knowledge about antibiotics [11], and different cultural orientations, with a more collectivist culture in Bulgaria with low social and institutional trust, a more individualistic culture and high trust in Sweden, with Italy in an intermediate position [12–14].

## Methods

### Design

**Fig. 1:**
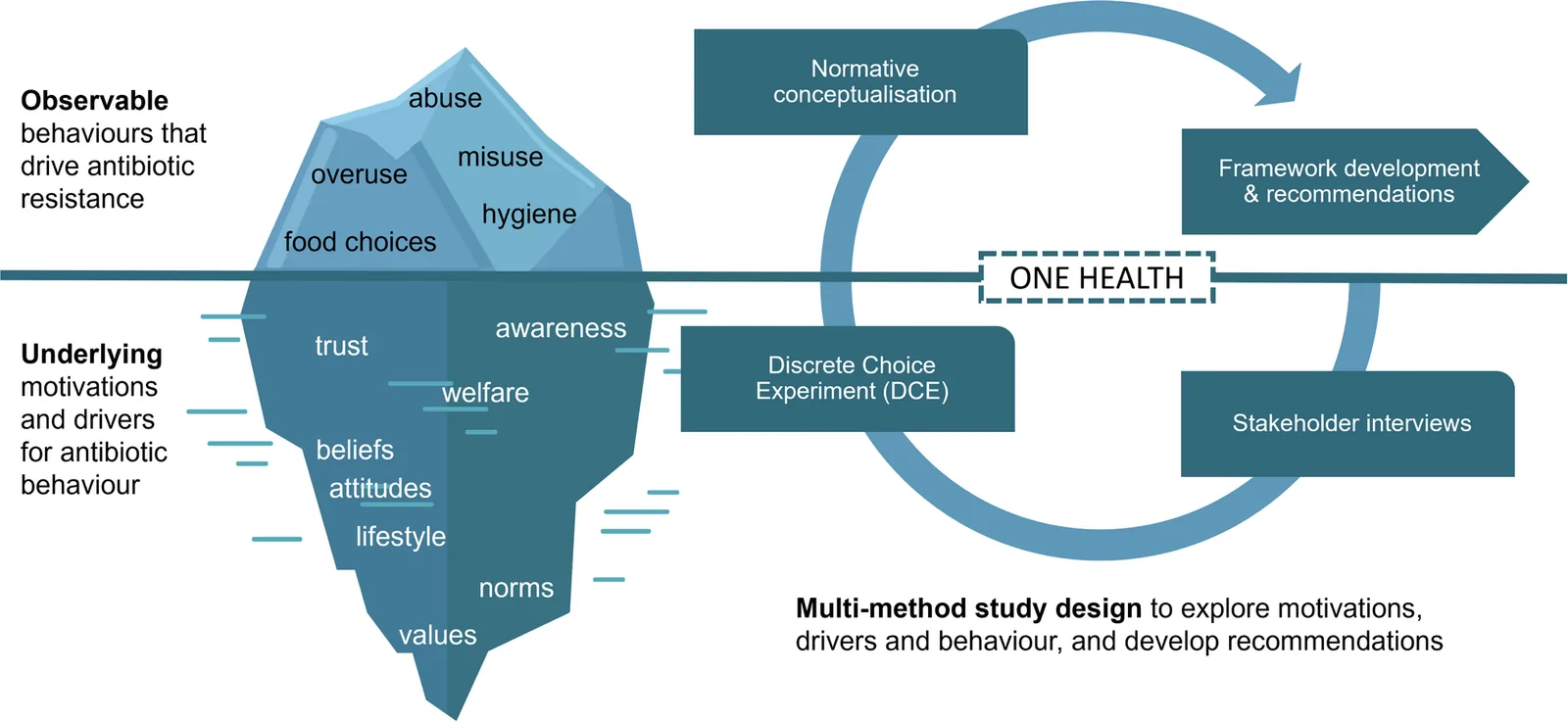
Project design and conceptual approach

This ongoing project (May 2025-April 2028) follows a comparative mixed-methods design and includes qualitative, quantitative, and normative components in Sweden, Italy, and Bulgaria. The qualitative component consists of interviews with stakeholders involved in the clinical use, procurement, supply, regulation, and governance of antibiotics. Findings from the stakeholder interviews, together with evidence from relevant literature and previous research [15–18], will inform the development of a survey incorporating a DCE that will be sent to members of the general public in the three countries. Normative analysis will be carried out alongside the empirical studies and will bring together the qualitative and quantitative findings through a reflective-equilibrium process. The combined findings will inform a context-sensitive methodological research framework and recommendations for antibiotic stewardship, policy, and communication. Figure 1 provides an overview of the project design and conceptual approach. The figure illustrates how observable behaviours relevant to AR may be shaped by less visible factors, including awareness, trust, beliefs, attitudes, norms and values. These factors are examined through the stakeholder interviews and survey, and integrated into the normative analysis and framework development, within a One Health perspective.

### Stakeholder interviews

#### Study design and conceptual framework

The qualitative step consists of a comparative study of semi-structured online interviews with 16–20 participants from four stakeholder groups in each country, including healthcare professionals, health system procurers, pharmaceutical industry and market-level representatives, and regulatory and public-agency officials. Interviews aim to capture how stakeholders across different sectors conceptualise the value of sustainable access to effective antibiotics, and justify actions and trade-offs intended to secure access in Sweden, Italy, and Bulgaria

The study follows an abductive logic, combining a theoretically informed approach grounded in the 3D Value Framework with openness to emerging themes [19]. The framework distinguishes three dimensions of value, namely patient value, population value, and preparedness value, which are being used to guide data collection and analysis. The framework guided the development of the interview guide, which was drafted collaboratively with experts in the 3D Value Framework and antibiotic innovation and informed by relevant literature and the study objectives. The guide explores how stakeholders understand the value of antibiotic access, what they perceive to be at stake when access is uncertain, and which actions, efforts, or trade-offs they consider justified. At the same time, the analysis remains open to themes that emerge from the empirical material. The interview guide was tested in pilot interviews involving one participant from each stakeholder group in each country.

#### Data collection 

Participants are being recruited through professional networks, associations, and institutional contacts. Recruitment and interviews are expected to be completed in November 2026. Inclusion criteria are professional activity in one of the identified categories, age over 18 years, and capacity to provide informed consent. Interviews are conducted online through secure, GDPR-compliant platforms. Potential participants receive written information about the study and the main interview questions in advance. At the beginning of each interview, verbal informed consent is obtained. Consent covers participation, audio recording, pseudonymised transcription, and the use of pseudonymised quotations. Interviews are audio-recorded with participants’ consent and transcribed verbatim

#### Data analysis

At the national level, transcribed interviews are analysed using an abductive qualitative approach informed by the 3D Value Framework. Local researcher teams will analyse transcripts in the national languages. Codes will be developed through a combination of framework-informed and data-driven coding

Following the national analysis, data from Sweden, Italy, and Bulgaria will be compared in a *transnational study* with transcripts translated into English for comparison and accessibility. The comparative analysis will draw on framework analysis [20], with a joint thematic framework combining categories emerging from the national analyses, in turn analysed to identify cross-country commonalities and differences in stakeholders’ reasoning and value justifications.

### Discrete choice experiment (DCE)

The DCE survey will examine the trade-offs respondents make in different choice tasks and explore how values, norms, and behavioural and sociocultural factors determine the public’s antibiotic behaviour. The DCE will operationalise input from literature and qualitative analysis of stakeholder interviews. Participants will be asked to imagine having a bacterial infection and be informed by their physician that they are in need of antibiotics to avoid complications. Depending on the final survey design, respondents may be allocated to scenarios in which the collective or personal dimensions of antibiotics are emphasised, including on the basis of prosociality scores. The survey will end with a quality-assurance section, including feedback questions to assess any difficulties experienced, the length of the questionnaire, and an invitation to provide additional feedback and comments.

DCE methodology is based on Random Utility Theory, presenting choice tasks that are used to elicit preferences, enabling analysis of the relative importance of different attributes [21]. This method is increasingly used to study healthcare settings [22]. Stakeholders in each country will be involved in drafting the survey. The questionnaire will cover sociodemographic and background questions, together with questions to test antibiotic knowledge, prosociality, trust, biospherism, gender-related perspectives, social norms, values, and salient themes emerging from the literature and stakeholder interviews. DCE choice tasks will be created using a Bayesian D-efficient design to estimate a standard multinomial logit model based on a main-effects utility function.

####  Pilot and data collection 

Pre-testing with peer debriefings and think-aloud methods will be followed by pilot-testing in each country. Inclusion criteria for the pilot and final survey: representatives of the general public, aged 18 or over, proficient in their national languages. Pilot testing is expected to test understanding of wording, instructions and choice tasks. After pilot testing, the survey will be simultaneously launched to a representative sample of the general population in Sweden, Italy, and Bulgaria. The sample size will depend on the final design [23], with a minimum of 350 respondents from each country

####  Data analysis

All variables will be analysed using descriptive statistics. The models used to analyse the DCE choice data will be selected according to the final choice-task structure and model fit. Data from each country will initially be analysed separately using a multinomial logit model, and the Swait-Louviere test will be used to assess scale differences between country samples [24]. Depending on model fit and data structure, latent class models or panel mixed multinomial models will be employed to estimate attributes and assess their relative importance

### Normative analysis and integration

Normative analysis will be carried out in connection with both the qualitative (interviews) and quantitative (DCE) results to provide normative input for a reflective equilibrium exercise. This empirical ethics approach combines normative analysis and empirical data to arrive at context-sensitive and specific ethical norms that guide action [25]. The process will be repeated at the end of the project, synthesising results into actionable recommendations for different actors.

####  Data analysis

Values and norms captured in empirical studies will be analysed with a method that strives towards a ‘reflective equilibrium’ between particular cases and general normative positions and principles [26]. In the first step, the stakeholder interviews will be analysed to identify values, responsibilities, and trade-offs that arise in professional and institutional reasoning about antibiotic access, stewardship, and preparedness. In the second step, the DCE will provide empirical input on how members of the public prioritise selected aspects of antibiotic treatment, responsible behaviour, and sustainable access. These empirical findings will not be treated as directly determining normative conclusions, but as material to be interpreted together with ethical principles and theories. In the final integrative step, the values and norms emerging from the empirical studies will be considered in relation to social and ethical theories of justice, personal and collective responsibility, and One Health. The 3D Value Framework will support this analysis by helping to identify which dimensions of antibiotic value are at stake, while the reflective-equilibrium process will be used to examine how these values should be interpreted, balanced, and translated into context-sensitive recommendations [27]

## Ethics and data management

Ethical approval was obtained in all three participating countries. In Sweden, the study was approved by the Swedish Ethical Review Authority (registration no. 2025-06647-01). In Italy, it was approved by the Eurac Research Ethics Review Board (registration no. 203305). In Bulgaria, it was approved by the Ethics Committee of Medical Center “NM Genomix” at its regular meeting held on 17 December 2025.

The study involves adult participants only. Participants in the qualitative interviews receive written information about the study before participation and provide informed consent before the interview. Interviews are audio-recorded with participants’ consent, transcribed, and pseudonymised. Survey participants will receive information about the study before taking part and will provide consent before completing the questionnaire.

Data will be handled in accordance with applicable data protection regulations, including the General Data Protection Regulation (GDPR). Personal data will be minimised, stored securely, and accessible only to authorised members of the research team. For the comparative analysis, relevant qualitative material will be translated into English, with identifying details removed or reduced where needed.

## Outputs and dissemination

The project will produce comparative empirical findings from the stakeholder interviews and the DCE survey, together with an integrative normative analysis of values, responsibilities, and trade-offs related to antibiotic behaviour, stewardship, and sustainable access.

These findings will inform a context-sensitive methodological framework for studying values, norms, and behavioural and sociocultural determinants of antibiotic resistance across national contexts. The framework will include guidance on how the study design can be adjusted to different national contexts and to support transnationality through translatability and generalisability, conceptualising commonalities and differences in a synthetic, holistic way [28]. The framework and recommendations will be discussed in stakeholders’ roundtables (national and international colleagues, AMR patient advocates and organisations, healthcare professionals, policy-makers, etc.) and improved before release [29].

## Discussion

This protocol presents a comparative mixed-methods empirical ethics study of antibiotic behaviour, stewardship, and sustainable access in Sweden, Italy, and Bulgaria. The project starts from the idea that responsible antibiotic behaviour cannot be understood only as a matter of individual knowledge or compliance with medical advice. It also depends on how antibiotic access, stewardship, supply, procurement, and preparedness are organised and justified. For this reason, the project combines stakeholder interviews, a public preference survey, and normative analysis. The stakeholder interviews examine how actors in clinical, procurement, market, regulatory, and governance positions reason about sustainable access. The DCE survey will investigate how members of the public prioritise selected trade-offs. Normative analysis will then be used to interpret these findings in relation to responsibility, justice, and context-sensitive antibiotic stewardship.

The project responds to the need for stronger humanities and social science contributions to antibiotic resistance research [30]. The protocol builds on previous work showing that antibiotic-related behaviour cannot be understood as a simple problem of knowledge or awareness. Earlier qualitative studies have shown that members of the public may recognise antibiotic resistance as a shared problem, while still finding it difficult to understand what individual responsibility requires in practice [10, 31]. Work on consumer behaviour has also shown that responsibility for antibiotic resistance can extend beyond clinical antibiotic use and include everyday practices, such as food consumption, that are shaped by habits, institutions, and available options [32]. The present project extends this line of work by connecting public perspectives with stakeholder reasoning and normative analysis. A central feature of the design is the link between the qualitative and quantitative components. Stakeholder interviews will inform the development of DCE attributes and scenarios, so that the survey reflects problems and trade-offs identified within the antibiotic system. The DCE will then examine how members of the public respond to selected questions concerning antibiotic treatment, responsible behaviour, and sustainable access. These empirical findings will be interpreted through an empirical ethics approach, bringing ethical theories of justice, personal and collective responsibility, and One Health into dialogue with the data. This is important because empirical findings can show how people reason and what they prioritise, but they do not by themselves determine what ought to be done.

The study also has limitations. Translating stakeholder reasoning into DCE attributes will require simplification, and some ethically relevant considerations may be difficult to represent in choice tasks. Cross-country comparison also depends on careful translation and interpretation of concepts that may not have identical meanings in Sweden, Italy, and Bulgaria. Finally, the study focuses on selected countries and stakeholder groups. The resulting framework will therefore need further adaptation and testing before being applied in other settings.

## Data Availability

No datasets were generated or analysed during the current study.

## Abbreviations

AMR: Antimicrobial resistance
AR: Antibiotic resistance
DCE: Discrete choice experiment
GDPR: General Data Protection Regulation
WHO: World Health Organization
